# Prolonged diarrhea among under-five children in Bangladesh: Burden and risk factors

**DOI:** 10.1371/journal.pone.0273148

**Published:** 2022-10-03

**Authors:** Md. Iqbal Hossain, A. S. G. Faruque, Monira Sarmin, Mohammod Jobayer Chisti, Tahmeed Ahmed

**Affiliations:** Nutrition and Clinical Service Division, International Centre for Diarrhoeal Disease Research, Bangladesh (icddr,b), Dhaka, Bangladesh; Universita degli Studi di Parma, ITALY

## Abstract

**Introduction & background:**

Prolonged (duration >7 to 13 days) diarrhea (ProD) in under-five children is a universal health problem including Bangladesh. Data on epidemiology and associated or risk factors of ProD are limited, particularly in Bangladesh where a high burden of ProD is reported. This study intended to assess the case load of ProD and its associated or risk factors compared to acute diarrhea (AD, duration ≤7 days).

**Methods:**

We analyzed the data collected between 1996–2014 from a hospital-based Diarrheal-Disease-Surveillance-System (DDSS) in the ‘Dhaka Hospital’ of International Centre for Diarrhoeal Diseases, Bangladesh (icddr,b). The DDSS enrolled a 2% systematic sample, regardless of age, sex, and diarrhea severity. The data included information on socio-demographic factors, environmental history, clinical characteristics, nutritional status, and diarrhea-pathogens. After cleaning of data, relevant information of 21,566 under-five children were available who reported with ≤13 days diarrhea (including AD and ProD), and their data were analyzed. Variables found significantly associated with ProD compared to AD in bi-variate analysis were used in logistic regression model after checking the multicollinearity between independent variables.

**Results:**

The mean±SD age of the children was 14.9±11.7 months and 40.4% were female; 7.6% had ProD and 92.4% had AD. Age <12 months, mucoid- or bloody-stool, warmer months (April-September), drug used at home before seeking care from hospital, and history of diarrhea within last one month were found associated with ProD (p<0.05); however, rotavirus infection was less common in children with ProD (p<0.05). ProD children more often needed inpatient admission than AD children (14.4 vs. 6.3, p<0.001). Case fatality rate of ProD vs. AD was 0.3% (n = 5) vs. 0.1% (n- = 22) respectively (p = 0.051).

**Conclusion:**

A considerable proportion (7.6%) of under-five children reporting to icddr,b hospital suffered from ProD. Understanding the above-mentioned associated or risk factors is likely to help policy makers formulating appropriate strategies for alleviating the burden and effectively managing ProD in under-five children.

## Introduction

About 80% of the deaths from diarrhea among children happen in the African and South-East Asian region including Bangladesh [1, 2]. Even with the progress in the management of diarrhea, it is still responsible for a death toll of about 525,000 deaths per year in under-five children [3]. Acute diarrheas are usually subsided or cured within seven days, but prolongation of duration of diarrhea between 7 and 13 days may be called as prolonged diarrhea (ProD) [4], and > 13 days as persistent diarrhea (PD). Although most of the diarrheal illnesses are acute (< 7 days duration termed as acute diarrhea, AD) in nature, the burden and risk factors of ProD [5] are less understood health problems worldwide among infants and young children including Bangladesh. Children with ProD or PD suffer from long-term hurdles such as malabsorption, malnutrition and growth failure and have a higher risk of death [6, 7]. An observation of 1,276 child-years reported that ProD accounted for significant morbidity and was a key predictor for PD, 11.7% of diarrheal episodes, and 25.2% of all days of diarrhea [4]. When a child’s diarrheal episode progressed from AD to ProD, the overall risk that the episode would evolve to PD increased from 4.8% to 29%. They also revealed significant decreases in weight-for-age z-score (-0.19 to -0.45, *p* = 0.0001) and height-for-age z-score (-0.81 to -1.40, *p* = 0.0002) in the period after children’s first ProD illness. Data on epidemiology and differential clinical features of ProD are inadequate, particularly in Bangladesh where the case load of different types of diarrhea is very high, and this information will be helpful in the proper management of ProD and interrupt its progression to persistent diarrhea to reduce and control the morbidity and mortality from these conditions. This study aimed to assess the case-load of ProD and its associated factors as well as to understand its differential features.

## Methods

We analyzed the data after extracting related information from the database ‘Diarrheal-Disease-Surveillance-System (DDSS)’ of the ‘Dhaka Hospital’ of International Centre for Diarrhoeal Diseases, Bangladesh (icddr,b). Dhaka Hospital of icddr,b; located in Dhaka, the capital city of Bangladesh provides care and treatment to people with diarrheal diseases since 1962. This hospital serves around 150,000 patients annually. The detailed description of the study site has been provided elsewhere [8]. All children admitted to this study hospital received standard management following the hospital guidelines. Dehydration was assessed and corrected accordingly by the modified WHO method (Dhaka method) which is almost similar to the World Health Organization (WHO) method and approved by the WHO. In addition to ORS, all children received zinc tablet. Antibiotics were prescribed only if indicated.

During the study period DDSS enrolled a 2% systematic sample, regardless of age, sex, and diarrhea severity. All related information was collected by research assistants by administering a structured questionnaire. The data included information on socio-demographic factors, clinical characteristics, nutritional status, and diarrhea pathogens. After cleaning of data, relevant information of 21,566 under-five children were available who reported with ≤13 days diarrhea (including AD and ProD), and their data were analyzed comparing ProD group with AD group.

### Statistical analysis

For normally-distributed continuous variables, means were compared using student’s t-test. Variables not normally distributed were compared by the Mann-Whitney U test. The differences in proportions were compared by chi-square test or Fisher’s exact test if the expected number in any cell was ≤ 5. A probability of less than 0.05 was considered statistically significant. The strength of association of variables associated with ProD compared to AD was determined by estimating odds ratios (ORs) and their 95% confidence intervals (CIs). Variables found significantly associated with ProD compared to AD in bi-variate analysis were included in logistic regression models. We checked the multicollinearity between independent variables using variance inflation factor (VIF). In the final model, the VIF values of all independent plausible variables were less than 2.

### Ethics

The aforementioned DDSS is approved by the Institutional Review Board of icddr,b composing i) Research Review Committee (RRC) and ii) Ethical Review Committee (ERC). Before data collection informed verbal consent from the participants was taken. Confidentiality was assured and maintained. The verbal-consent process included explanation of the research process and what the participant would do if she or he agreed to participate in the study and took place between the research assistant and the likely participant in presence of a witness. For the documentation of verbal consent a check mark is given in the questionnaire, and this consent was acknowledged by the participants. No individual identifying information was used for reporting and publication purposes.

## Results

**Fig 1:**
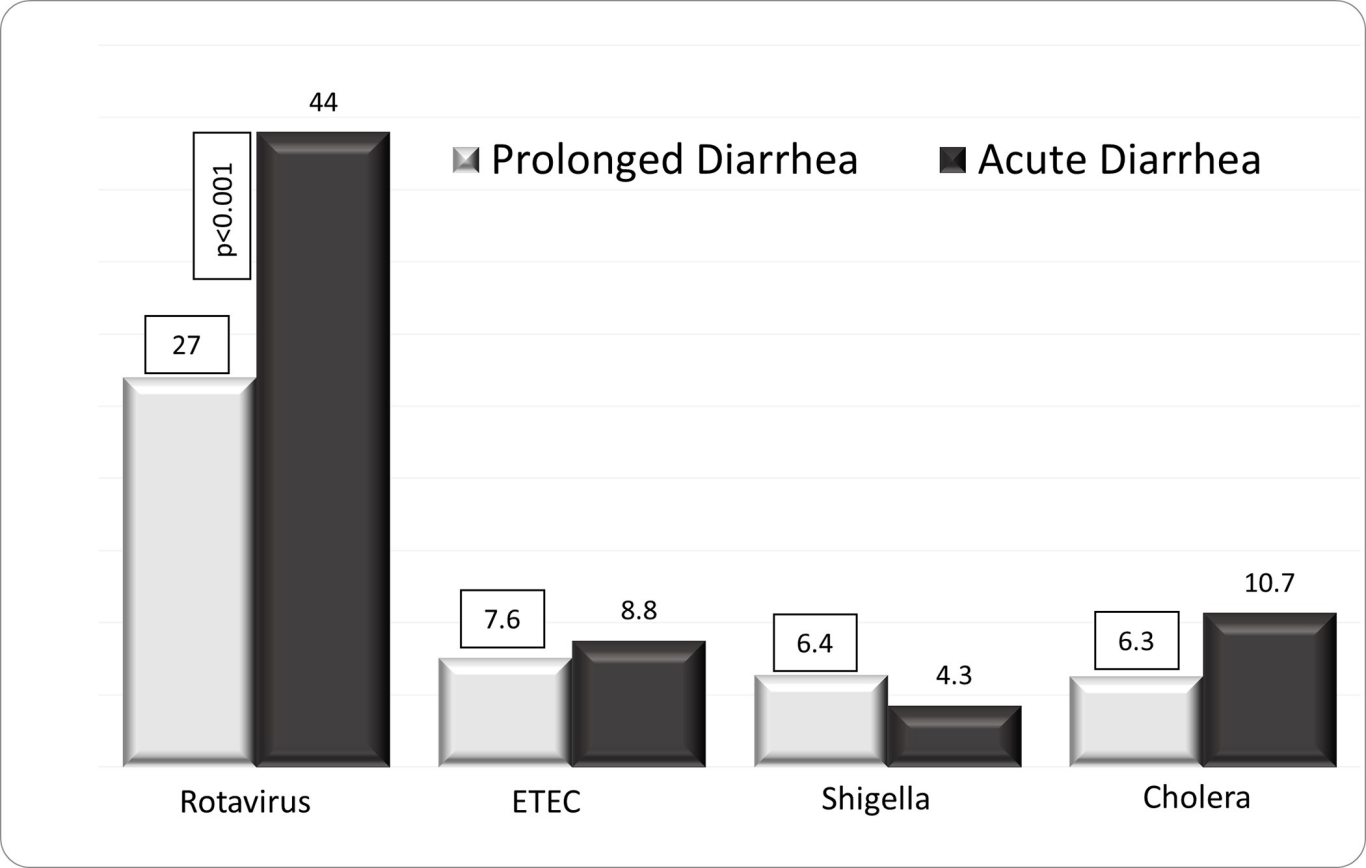
Percent of children with different stool organisms by prolonged diarrhea and acute diarrhea.

**Fig 2:**
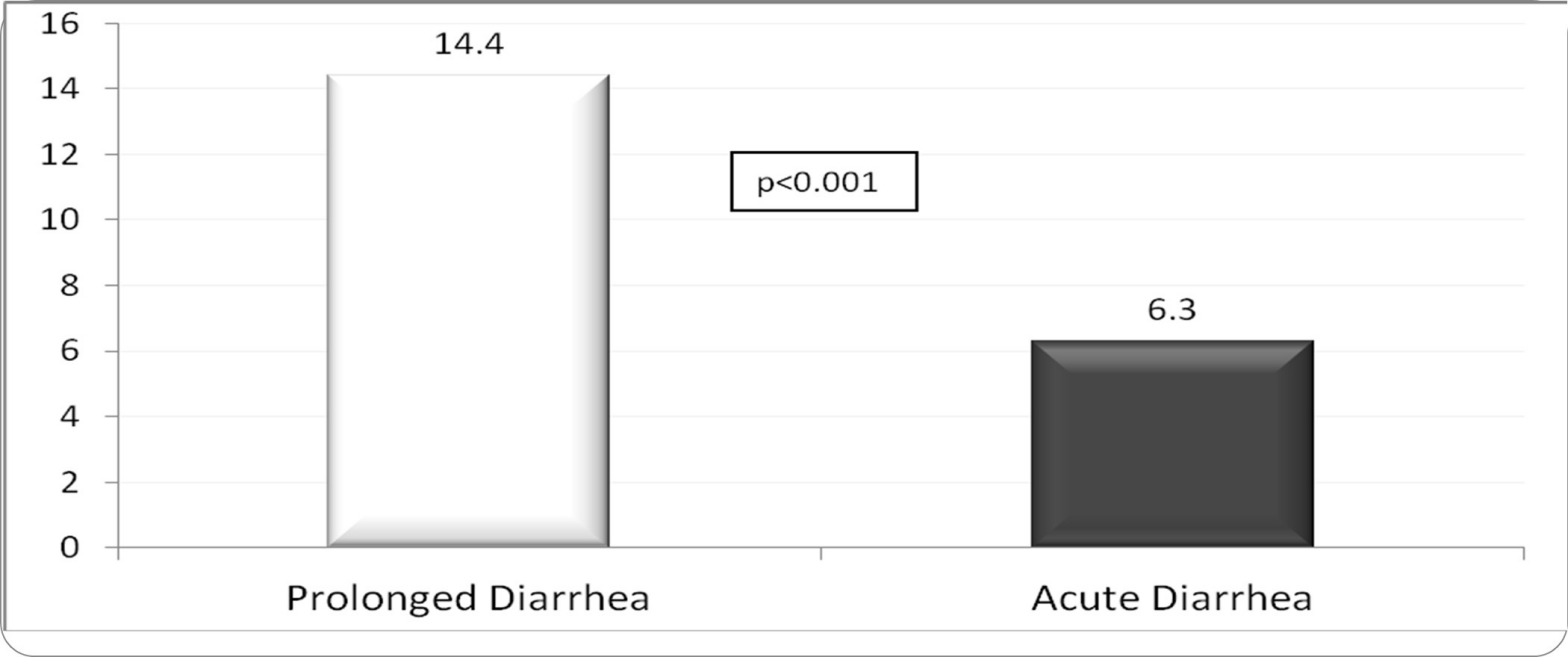
Percent of children required inpatient admission.

**Fig 3:**
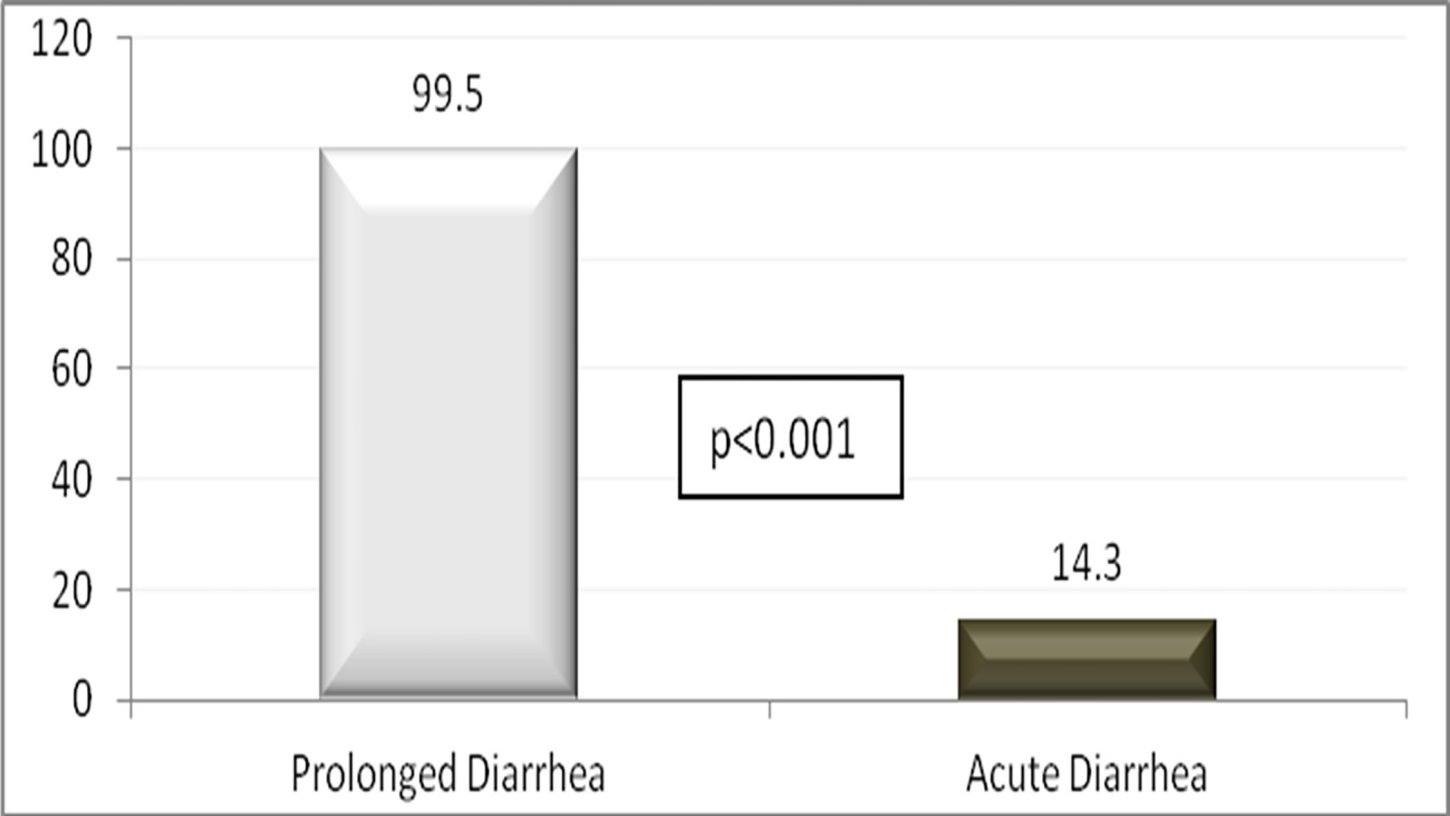
Percent of children with prolonged diarrhea and acute diarrhea required hospital stay for >3 days.

**Fig 4:**
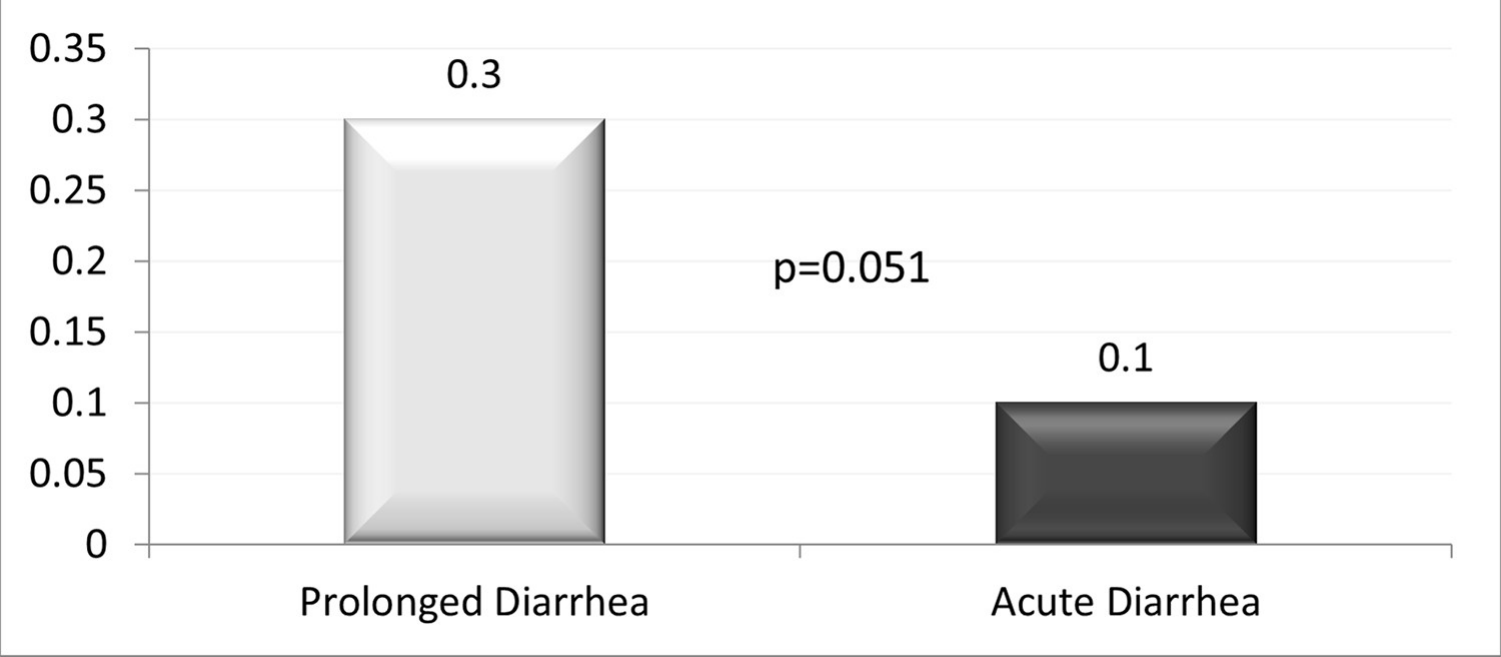
Case fatality rate (%) of children with prolonged diarrhea and acute diarrhea.

**Fig 5:**
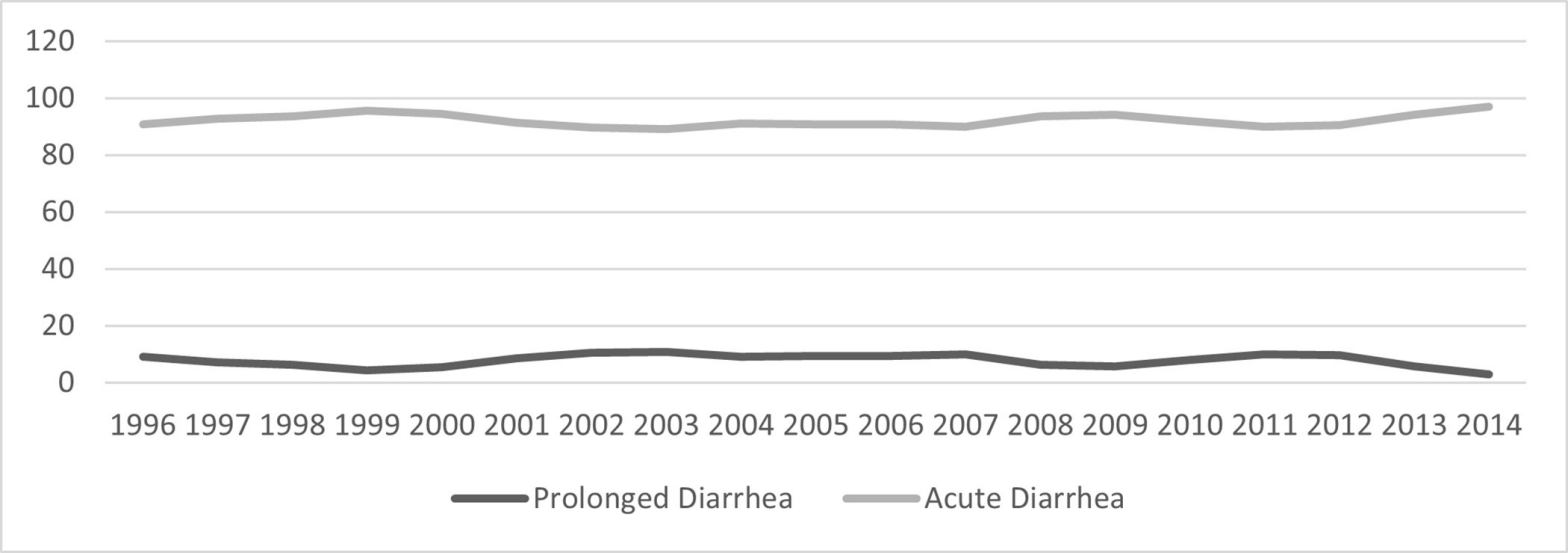
Children (%) with prolonged diarrhea and acute diarrhea by year.

**Fig 6:**
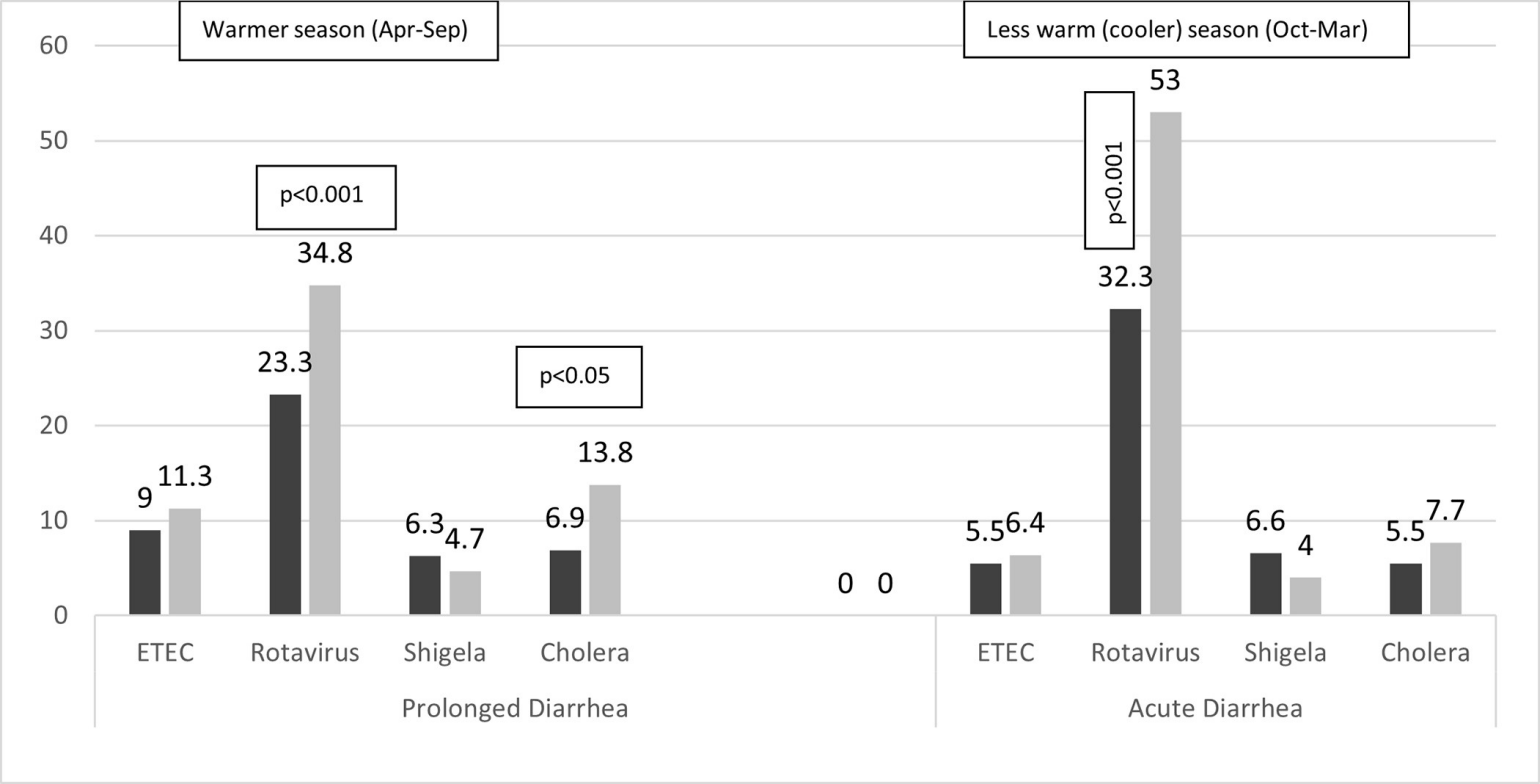
Stool isolates (%) in children with prolonged and acute diarrhea by warmer or less warm/cooler season.

The (mean±SD) age of the children was 14.9±11.7 months, 40.4% were girl, and 1645 (7.6%) had ProD. Detailed results of bivariate analysis is shown in Table 1. Age <12 months, mucoid or bloody stool, presenting during warmer months (April-September), receiving drug before reporting to hospital, and having history of diarrhea within last one month were found to be associated with ProD (p<0.05) (Table 2); however, rotavirus infection was less common in children with ProD (p<0.05) (Table 1 and Fig 1). Sex of the child, stool frequency, dehydration status, abdominal pain, breast feeding and/or nutritional status, parental education or socioeconomic condition were found similar in frequency between the children suffering from ProD and AD (Table 2). Also, similar rates of infection with *E*. *coli* (ETEC) 8.7%, shigella 4.5% and cholera 10.4% were observed between the two groups of children (Table 2 and Fig 1). Children with ProD more often required inpatient admission than those with AD (14.4 vs. 6.3, p<0.001) (Figs 2 and 3). Case fatality rate of ProD vs. AD was 0.3% (n = 5) vs. 0.1% (n = 22) respectively (p = 0.051) (Fig 4). The proportion of children admitted with ProD and AD during the study period was similar per year (Fig 5). Fig 6 shows the organism isolated from stool sample of the children suffering from ProD and AD during warmer or cooler months. During warmer months cholera was more common in ProD. However, during warmer- and cooler- months rotavirus was less common in children with ProD.

**Table 1:** Differential features of under-five children with prolonged diarrhea (ProD) (diarrhea for > 7 to 13 days) and acute diarrhea (AD) (diarrhea for ≤ 7 days).

Variable	ProD N = 1645 n (%)	AD N = 19921 n (%)	p-value
Infant (age ≤ 12 months)	1143 (69.6)	11884 (59.7)	<0.001
Female	648 (39.4)	8064 (40.5)	0.403
Stool frequency > 10 during last 24 hours	673 (40.9)	8717 (43.8)	0.013
Mucoid or bloody stool	638 (38.8)	4294 (21.6)	<0.001
No abdominal pain	493 (30.0)	4782 (24.0)	<0.001
No dehydration	1151 (70.0)	13101 (65.8)	<0.001
Fever on admission	21 (4.3)	358 (6.2)	0.112
Edema	12 (0.7)	77 (0.4)	0.038
Wasting (WHZ < -2)	516 (31.4)	4983 (25.0)	<0.001
Stunting (HAZ < -2)	588 (35.7)	6486 (32.6)	0.004
Providing other than breast milk before 6 months of child’s age (data among > 6 months age group)	966 (78.0)	11095 (72.1)	<0.001
Did not receive vitamin A supplementation within last 6 months age (among > 12 months age group)	163 (32.5)	2554 (31.8)	0.767
During warmer months (April-September)	975 (59.3)	9908 (49.7)	<0.001
Received drug before reporting to hospital	228 (47.4)	1450 (18.3)	<0.001
Using untreated water for drinking purpose	1096 (66.6)	12901 (64.8)	0.133
Illiterate mother	581 (35.3)	5893 (29.6)	<0.001
Illiterate father	520 (33.5)	5571 (28.0)	0.001
Residence in slum	167 (10.2)	1738 (8.7)	0.027
Mother works out of home	175 (10.7)	2233 (11.3)	0.488
Mother’s age < 20 to or > 40 years	130 (7.9)	1591 (7.7)	0.703
Poor (lower two asset quintiles)	651 (39.7)	7331 (36.8)	0.011
Used non-sanitary latrine	618 (37.6)	6624 (33.3)	<0.001
Had previous episode of diarrhea in last 1 month	172 (10.5)	1583 (8.1)	<0.001
Had history of cough and fever in last 1 month	1199 (72.9)	13926 (69.9)	0.006
Non-Muslim	58 (3.5)	744 (3.7)	0.729
Rotavirus positive stool	440 (27.0)	8632 (44.0)	<0.001
ETEC positive stool	124 (7.6)	1751 (8.8)	0.086
Shigella positive stool	105 (6.4)	866 (4.3)	<0.001
Cholera positive stool	104 (6.3)	2138 (10.7)	<0.001

**Table 2:** Associated or risk factors of prolonged diarrhea (ProD) (diarrhea for > 7 to 13 days) and acute diarrhea (AD) (diarrhea for ≤ 7 days) by logistic regression (enter method).

	p-value	Adjusted Odds ratio	95% C.I.	
			Lower	Upper
**Infant (age ≤ 12 months)**	**<0.001**	**1.828**	1.436	2.328
Stool frequency > 10 during last 24 hours	0.725	0.959	0.759	1.212
**Mucoid or bloody stool**	**<0.001**	**1.906**	1.480	2.456
No abdominal pain	0.059	1.291	0.990	1.683
No dehydration	0.915	1.015	0.773	1.333
Providing other than breast milk before 6 months of child’s age (data among > 6 months age group)	0.906	1.017	0.771	1.341
Edema	0.547	1.616	0.339	7.712
Wasting (WHZ < -2)	0.089	1.258	0.965	1.639
Stunting (HAZ < -2)	0.536	1.082	0.844	1.387
**During warmer months (April-September)**	**0.017**	**1.323**	1.051	1.664
**Received drug before reporting to hospital**	**<0.001**	**4.405**	3.465	5.601
Illiterate mother	0.387	1.145	0.843	1.554
Illiterate father	0.203	0.814	0.594	1.117
Residence in slum	0.126	1.350	0.919	1.983
Poor (lower two asset quintiles)	0.084	1.288	0.967	1.717
Used non-sanitary latrine	0.400	1.120	0.860	1.460
**Had previous episode of diarrhea in last 1 month**	**0.041**	**1.405**	1.014	1.947
Had history of cough and fever in last 1 month	0.192	0.849	0.663	1.086
**Rotavirus positive stool**	**<0.001**	**0.396**	0.301	0.519
Shigella positive stool	0.168	0.730	0.467	1.141
Cholera positive stool	0.163	0.743	0.490	1.127
Constant	<0.001	0.020	-	-

## Discussion

In this case-control study of a large number of children reporting to the world’s largest diarrheal disease treatment hospital, we found that ProD accounts for significant morbidity. It is associated with younger age, mucoid or bloody stool, warmer month, receiving drug prior to reporting to hospital, and having a history of diarrhea within last one month. Furthermore, we showed that ProD children more often needed inpatient admission and case fatality rate of ProD was higher than that of acute diarrhea.

The observation of association of younger age with ProD might be due to development of lesser immunity against infectious disease including diarrhea during infancy [9] compared to their elder counterparts. Mucoid or bloody stool is associated with infection and inflammation of the intestine and usually take longer time than toxin induced acute watery diarrhea. Drugs received by the patients prior to hospitalization could not be specified by the guardian(s) attending the children and that might not be appropriate for the nature of diarrhea, thus might alter the intestinal flora that might further lead to mal-absorption followed by ProD. History of having diarrhea within last one month might leave the memory of recent insult in the intestinal flora of affected children that potentially led to delay in re-epithelization of intestinal flora and prolong the acute episode of diarrhea. Although a number of studies/reports related to infectious diarrhea and/or food poisoning- temperature may directly or indirectly influence the replication and survival of pathogens that cause diarrhea. We do not have any ready explanation for the association of warmer month with ProD, however, we may speculate that warmer season might have some environmental factors that potentially interrupt natural intestinal re-epithelization leading to continuation of diarrhea beyond seven days.

The observation of higher case fatality rate among the children with ProD compared to those with AD is not surprising. Previous studies [6, 7] revealed that children with ProD suffer from mal-absorption followed by malnutrition potentially leading to fatal outcome.

Heavy burdens of diarrhea and undernutrition in early childhood seriously impair growth and development [10]. As mentioned before a significant proportion (up to 20%) of illness with AD progress into ProD or PD [6], and results in prolongation of the morbidity and malnutrition. Malabsorption, malnutrition and growth failure are common consequences and they have a higher risk of death [6, 7]. ProD can play a key role in the vicious cycle of childhood diarrhea and malnutrition. Thus, the identification of effective, affordable and acceptable interventions for ProD management remains an important challenge and there is an immediate need to appropriately manage ProD in order to interrupt the progression to PD, which adversely impact on diarrhea morbidity and mortality. Knowing these factors would be helpful to prevent and control these important consequences.

Existence of a sampling bias cannot be excluded in this study, as the children presenting to a large hospital system specialized in diarrheal illness likely do not have shorter, acute self-resolving episodes of diarrhea, and this might skew the results in favor of prolonged diarrhea. Since data on healthy controls were not available this could be one limitation in this study.

To our knowledge, this is the first study from South East Asia to examine ProD as a distinct class of diarrhea, thus our findings have several important public health implications that merit further consideration.
